# A Novel Strategy to Improve the Therapeutic Efficacy of Gemcitabine for Non-Small Cell Lung Cancer by the Tumor-Penetrating Peptide iRGD

**DOI:** 10.1371/journal.pone.0129865

**Published:** 2015-06-12

**Authors:** Qing Zhang, Yang Zhang, Ke Li, Haiyu Wang, Huizhong Li, Junnian Zheng

**Affiliations:** 1 Cancer Institute, Xuzhou Medical College, Xuzhou, Jiangsu, 221002, China; 2 Jiangsu Center for the Collaboration and Innovation of Cancer Biotherapy, Cancer Institute, Xuzhou Medical College, Xuzhou, Jiangsu, 221002, China; 3 Department of Oncology, Xuzhou Central Hospital, Xuzhou Clinical School of Xuzhou Medical College, Affiliated Hospital of Southeast University, Xuzhou, Jiangsu, 221009, China; Ospedale Pediatrico Bambino Gesu', ITALY

## Abstract

Non-small cell lung cancer (NSCLC) is the most common type of lung cancer, comprising approximately 75–80% of all lung cancers. Gemcitabine is an approved chemotherapy drug for NSCLC. The objective of this study was to develop a novel strategy to improve the therapeutic efficacy of Gemcitabine for NSCLC by the co-administered iRGD peptide. We showed that the rates of positive expression of αvβ3, αvβ5 and NRP-1 in the A549 cell line were 68.5%, 35.3% and 94.5%, respectively. The amount of Evans Blue accumulated in the tumor of Evans Blue+iRGD group was 2.5 times that of Evans Blue group. The rates of growth inhibition of the tumors of the iRGD group, the Gemcitabine group and the Gemcitabine+iRGD group were 8%, 59.8% and 86.9%, respectively. The results of mechanism studies showed that PCNA expression in the Gemcitabine+iRGD group decreased 71.5% compared with that in Gemcitabine group. The rate of apoptosis in the Gemcitabine+iRGD group was 2.2 time that of the Gemcitabine group. Therefore, the tumor-penetrating Peptide iRGD can enhance the tumor-penetrating ability and therapeutic efficacy of Gemcitabine in the A549 xenograft. The combined application of Gemcitabine with iRGD may be a novel strategy to enhance the clinical therapeutic efficacy of Gemcitabine in patients with NSCLC.

## Introduction

Lung cancer is the leading cause of cancer-related deaths worldwide and continues to show an increasing incidence [1, 2]. Non-small cell lung cancer (NSCLC) is the most common type of lung cancer, comprising approximately 75–80% of all lung cancers [3]. The diagnosis is frequently made in patients with advanced-stage disease, and in almost two-thirds of all cases, the cancer has already spread beyond a localized area at the time of diagnosis [4–6]. This limits the options for therapy and leads to a poor prognosis with a median survival of less than 12 months [7, 8]. Most patients with NSCLC present with unresectable disease, and therefore, the current treatment options of chemotherapy and radiotherapy are palliative at best [7, 9]. Many traditional cytotoxic drugs, including Vindesine, Carboplatin, Etoposide, Ifosfamide, Cyclophosphamide and Mitomycin, have been used as a monotherapy in cases of NSCLC. In the past few years, some new chemotherapeutic agents such as Vinorelbine, Paclitaxel, Docetaxel and Gemcitabine have also been applied to treat NSCLC. However, these chemotherapeutic agents only offer small improvements in overall survival [10].

Gemcitabine is a deoxycytidine analog that is converted in vivo into active metabolites including difluorodeoxycytidine di- and triphosphate [11]. The triphosphate analogue of Gemcitabine replaces one of the nucleic acid building blocks (in this case, cytidine) during DNA replication. This process arrests tumor growth and ultimately leads to apoptosis. Another target of Gemcitabine is ribonucleotide reductase. The diphosphate analogue binds to the active site of ribonucleotide reductase and irreversibly inactivates the enzyme. Once the enzyme is inhibited, DNA replication and repair are terminated, and apoptosis is induced [11–13]. Gemcitabine has been approved by the Food and Drug Administration (FDA) as a treatment for advanced and metastatic NSCLC, pancreatic cancer, bladder cancer, ovarian cancer and breast cancer alone or in combination with other drugs [14].

Clinical trials have demonstrated that Gemcitabine could prolong survival and improve the quality of life of patients with advanced NSCLC [14, 15]. Some studies have reported that Gemcitabine consistently yields response rates that exceed 20% when used as a single agent [14, 16, 17]. However, Gemcitabine often fails to achieve adequate disease control because of insufficient cytotoxicity to the tumor cells and intolerable side effects. The median survival is extended for just 2–4 months, and most patients die of disease progression [7, 9]. Therefore, a simple increase in the dose cannot improve the condition of patients; on the contrary, this may lead to serious side effects.

Previous studies have shown that the crossing of the vascular wall and the penetration into the tumor parenchyma against the elevated interstitial pressure in tumors remain a major challenge to the therapeutic efficacy of most clinical drugs [18]. Some anti-cancer drugs can only penetrate a distance of 3 to 5 cell diameters from the blood vessels in solid tumors [19]. For example, the concentration of Doxorubicin decreases exponentially as the distance from tumor blood vessels increases, and it reaches half of its perivascular concentration at a distance of approximately 40 μm [20]. The distribution of Trastuzumab (Herceptin) in the interior region of breast tumor xenografts is also highly heterogeneous, which results in a lack of exposure of many tumor cells to detectable levels of the drug [21]. Therefore, increased tumor permeability may be a useful approach to enhance the therapeutic efficacy of chemotherapy drugs.

iRGD is a tumor-penetrating peptide (CRGDK/RGPD/EC) that has a specific permeability in tumor tissues and in the tumor vasculature [22]. The RGD tripeptide of iRGD can bind the integrins αvβ3 and αvβ5, the expressions of which are largely restricted to tumors (including tumor vasculature and cells) [22, 23]. When iRGD binds to integrins, the peptide bond between the amino acids K and G is proteolytically cleaved by the cell surface-associated proteases; then, the cryptic C-end Rule (CendR) motif (CRGDK/R) is exposed. The CendR motif then binds to neuropilin-1 (NRP-1). The activation of NRP-1 increases the permeability of blood vessels and tumor tissues, which allows drugs to penetrate into the internal tissue of the tumor much more easily [22–24]. It has been confirmed that iRGD has valuable potential as a targeting probe for the molecular imaging of tumors and for drug delivery, and can enhance the efficacy of drugs up to 3-fold [24].

In this study, we combined Gemcitabine with iRGD to treat nude mice xenografts of human NSCLC established with the A549 cell line. Our results demonstrated that iRGD could effectively stimulate Gemcitabine to inhibit tumor cell proliferation and induce tumor cell apoptosis. The in vivo therapeutic efficacy was also significantly enhanced. These results suggested that combination therapy with iRGD may be a promising method to improve the clinical efficacy of Gemcitabine for the treatment of NSCLC.

## Materials and Methods

### Cell culture

The human NSCLC-derived cell line A549 was purchased from the Shanghai Institute of Biochemistry and Cell Biology, Chinese Academy of Sciences (Shanghai, China), and cultured in F-12K medium (Gibco, Grand Island, NY, USA) with 10% fetal bovine serum (Gibco, Grand Island, NY, USA) and 1% penicillin/streptomycin (Gibco, Grand Island, NY, USA).

### Flow cytometry

A total of 1 × 10^6^ A549 cells were incubated with fluorescent-labeled antibodies diluted in 100 μl phosphate-buffered saline (PBS) for 30 min at room temperature. The cells were then washed, suspended and evaluated with a FACS machine (FACSCanto II, Becton-Dickinson, USA). A matched isotype control antibody was used in all analyses. Finally, all the data were analyzed by FlowJo software (Tree Star, USA). FITC-conjugated mouse anti-human integrin αvβ3 antibody and integrin αvβ5 antibodies were purchased from Chemicon (CA, USA). The matched isotype control antibody, FITC-conjugated mouse IgG1κ, was purchased from eBioscience (San Diego, CA, USA). PE-conjugated mouse anti-human NRP-1 antibody and its isotype control were purchased from MACS Miltenyi Biotec (Bergisch Gladbach, Nordrhein-Westfalen, Germany).

### Mice and in vivo experiments

In vivo experiments involved 6 week-old female BALB/C nude mice (Vital River Laboratory Animal Technology Co., Ltd, Beijing, China), which were housed in the specific pathogen-free animal facility of Experimental Animal Center, Xuzhou Medical College (China). Animals were housed with a 12-hour light/dark cycle, in temperature (22+/-1°C) and humidity (55+/-5%) controlled room. All mice were allowed free access to sterile water and food. All cages housed up to 6 animals and contained wood shavings and an independent air supply system. All animal experimental protocols were approved and reviewed by the Institutional Animal Care and Use Committee of the Jiangsu Provincial Academy of Chinese Medicine (SCXK 2012–0005). During in vivo experiments, animals in all experimental groups were examined daily for physical activity. At the end of the experiment, mice were sacrificed by cervical dislocation.

### Tumor model

The human NSCLC model was established as previously described [25]. In brief, 1 × 10^7^ A549 cells were injected into the left forelimb armpits of 6 BALB/c nude mice. When the tumors grew to approximately 150 mm^3^, the tumors were harvested and segmented into tissue blocks (4–6 mm^3^ in size). Then, the left forelimb armpits of BALB/c nude mice were inoculated s.c. with the tissue blocks using a trocar. The mice were treated when the tumors grew to the desired size.

### Immunofluorescence staining

When the tumors reached approximately 200 mm^3^, mice were sacrificed, and the tumors were dissected and sectioned on a cryostat. The sections of the four groups (n = 3) were incubated overnight at 4°C with the same anti-human αvβ3, αvβ5 and their isotype control antibodies as those used in the flow cytometric analysis. The sections of the two groups were stained overnight at 4°C with a primary rabbit anti-human NRP-1 antibody (Abcam, Beverly, MA, USA) and its isotype control antibody (Abcam, Beverly, MA, USA). Then, the sections were stained with a DyLight 549-conjugated goat anti-rabbit IgG (H+L) secondary antibody (EarthOx, San Francisco, CA, USA) at 37°C for 1 hour. All sections were then observed and photographed with a fluorescence microscope (DS-Ri1, Nikon, Japan).

### Tumor permeability assay

When the tumors reached approximately 150 mm^3^, a total of 50 mg/kg Evans Blue, 4 mg/kg iRGD, 50 mg/kg Evans Blue + 4 mg/kg iRGD in 200 μl of PBS as vehicle, or 200 μl PBS alone were injected into the four groups of tumor-bearing mice (n = 3) respectively. Thirty minutes later, the mice were anesthetized by chloral hydrate and were perfused through the heart with PBS with 1% BSA. Then, organs and tumor tissues were collected. For Evans Blue quantification, 10 μl N,N-dimethylformamide was added for every 10 mg of tissue. After homogenization, the mixtures were incubated at 37°C for 24 hours, the mixtures were then centrifuged, and the supernatants were harvested. Finally, quantification was performed by measuring the absorbance at 600 nm with a spectrophotometer (BioTek Epoch, USA).

### In vivo efficacy studies

When the tumors reached approximately 100 mm^3^ after 10 days, the mice were divided into four groups (n = 6). In all, 100 mg/kg Gemcitabine (Merck, Darmstadt, Germany), 4 mg/kg iRGD, 100 mg/kg Gemcitabine + 4 mg/kg iRGD in 200 μl of PBS as vehicle, or 200 μl PBS alone were injected into the mice of the four groups via the tail vein. The injections were performed twice a week for a total of 8 times. The volume of the tumors was measured in two perpendicular directions with a caliper, and the weight of the nude mice was calculated every three days. The volume was measured using the following formula: V = 0.5 × (W^2^ × L), where V = tumor volume, W = the smaller perpendicular diameter and L = the larger perpendicular diameter. On day 30, mice were sacrificed, tumors were collected and weighed. The tumor growth inhibition ratio (TGIR) was calculated using the formula TGIR = (W − W_t_) / W × 100%, where W is the average tumor weight of the control group and W_t_ is the average tumor weight of the treatment group. All tumors were cut into two parts respectively, and were processed as following description.

### Immunohistochemical staining

One half of each tumor harvested at the end of the treatment study were fixed in 10% neutral-buffered formalin, then embedded in paraffin, and cut into 3–5μm sections [26]. The experiments were performed with a streptavidin-peroxidase system (SP) according to the manufacturer's protocol (ZSGB-BIO, Beijing, China). Proliferating cell nuclear antigen (PCNA) was detected with an anti-PCNA antibody (Abcam, Beverly, MA, USA). Imaging was performed by fluorescence microscopy (DS-Ri1, Nikon, Japan). To determine the IOD index of PCNA, five representative visual areas that were positive for PCNA were examined from each tumor. The PCNA index for each selected area was analyzed using IPP software.

### TUNEL assay

The paraffin sections were prepared as described above. The apoptotic cells were detected using a TdT-mediated dUTP nick end labeling (TUNEL) Kit according to the manufacturer's protocol (Roche, Mannheim, Germany). The number of TUNEL-positive cells was counted in five randomly selected fields from each tumor, and the apoptotic index for each field was calculated as the percentage of TUNEL-positive cells relative to 100 randomly selected cells.

### Western blot analysis

The other half of each tumor was snap-frozen in liquid nitrogen. Then, the total protein was extracted. PCNA was detected by normal Western blotting with the same anti-PCNA antibody as that used in the immunohistochemistry experiment. IRDye 800CW goat anti-rabbit IgG (H+L) antibody (LI-COR Biosciences, Lincoln, NE, USA) was used as the secondary antibody. β-actin was also detected as an internal control. Images were obtained by Odyssey (LI-COR, USA) and intensity quantifications of the Western blot bands were performed by ImageJ software (National Institutes of Health, USA).

### Statistical analysis

SPSS version 16.0 for Windows was used for all analyses. Quantitative data are presented as the mean ± standard deviation (SD); a comparison between two groups was performed by independent-sample t-test, while multiple samples were compared with one-way ANOVA, with α = 0.05 as a level for the test. Results were considered statistically significant at P < 0.05.

## Results

### Expression of NRP-1, ανβ3 and ανβ5 in the human NSCLC-derived cell line A549

**Fig 1:**
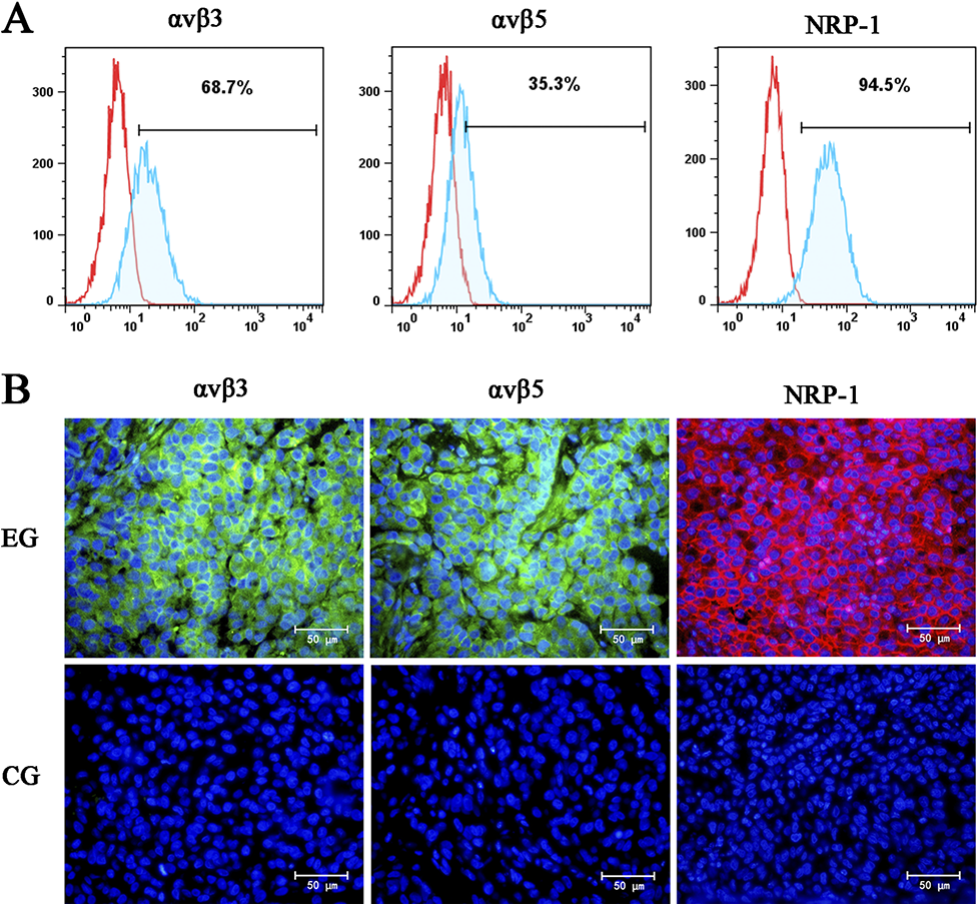
Expression analysis of αvβ3, αvβ5 and NRP-1 in A549 cells and xenografts. (A) Expression analysis of αvβ3, αvβ5 and NRP-1 in A549 cells by flow cytometry. (B) Expression analysis of αvβ3, αvβ5 and NRP-1 in A549 xenografts by immunofluorescence staining. EG, experimental group; CG, control group. Both αvβ3 and αvβ5 are stained green. NRP-1 is stained red. Nuclei are stained blue. Magnification, × 400; Scale bars = 50 μm.

The expression of integrins is largely restricted to tumors (including the tumor vasculature and cells), and other sites of angiogenesis or tissue repair [22, 27]. Neuropilin-1 is also overexpressed in many tumors [22, 28]. The tumor-penetrating ability of iRGD mainly depends on the molecules ανβ3, ανβ5 and NRP-1 overexpressed in cancer cells [18]. In order to confirm the expression of these molecules in the human NSCLC cell line A549, we performed a flow cytometric analysis. As shown in Fig 1A, the positive expression rates of αvβ3, αvβ5 and NRP-1 were 68.5%, 35.3% and 94.5%, respectively. This result demonstrated that the A549 cell line can be used to establish a human NSCLC model to study the effects of co-administered iRGD.

To confirm the expression of ανβ3, ανβ5 and NRP-1 in tumor tissue, we developed a BALB/c nude mouse xenograft model with the human NSCLC cell line A549. The expression of αvβ3, αvβ5, and NRP-1 was further detected by immunofluorescence staining. As shown in Fig 1B, αvβ3 (left), αvβ5 (middle) and NRP-1 (right) were overexpressed in the xenograft tissues.

### Tumor-penetration of Evans Blue co-administered with iRGD

**Fig 2:**
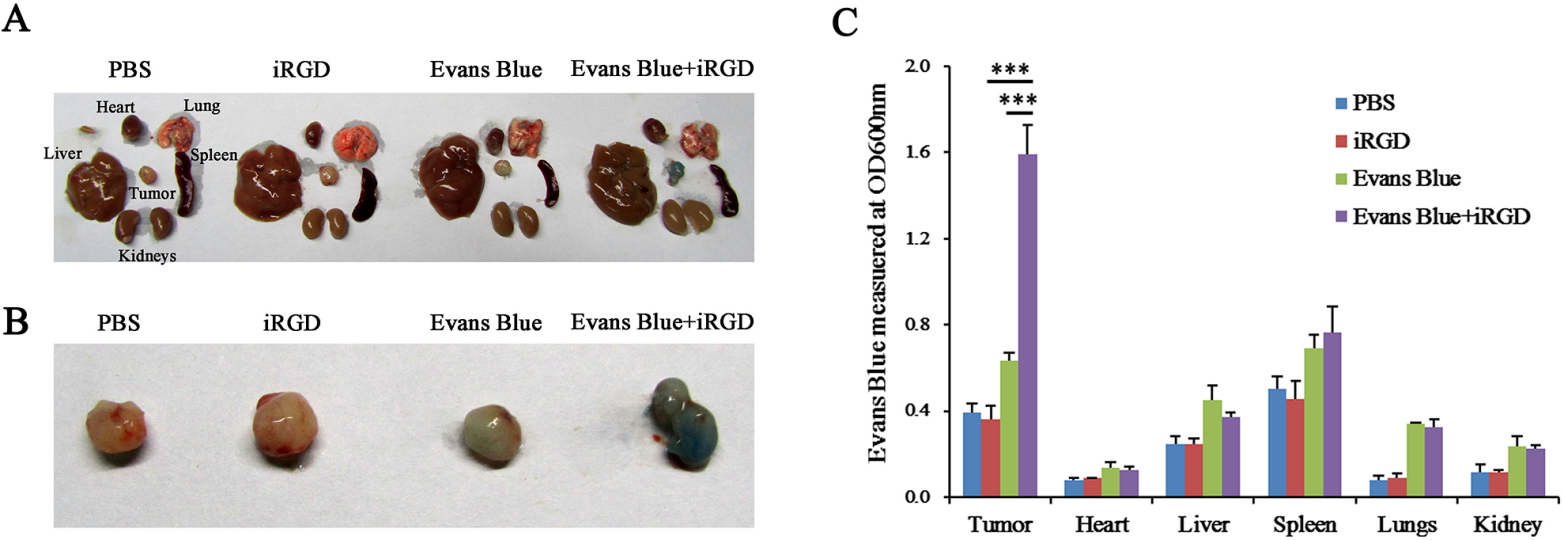
Penetration analysis of Evans Blue in extravascular tumor tissues. (A) The representative images of Evans Blue accumulation in normal organs and in tumors of the mice that received different treatments. The blue intensity denotes the quantity of accumulated Evans Blue. (B) Images of the tumor in (A). (C) Quantification of Evans Blue extracted from tumors and organs in (A) by OD600 measurement. n = 3; Error bars, mean ± SEM; ***p< 0.001.

To confirm the in vivo effect of the tumor-penetrating peptide iRGD, Evans Blue dye was used as a model drug to determine whether iRGD can promote the uptake of a drug into a tumor [18]. Evans Blue is an albumin-binding dye that can penetrate the organs via the blood and dye the organs blue. Compared with the controls, tumors of the Evans Blue+iRGD group were dyed a deeper blue, which was visible to the naked eye. However, the blue color of the normal organs, including the heart, liver, spleen, lung and kidney, was not obvious (Fig 2A and 2B). To further confirm that the effect of iRGD to specifically promote the uptake of Evans Blue dye into tumor tissue, the Evans Blue in the tumors and organs was extracted and quantitatively analyzed by spectrophotometry. The results showed that, in general, the amount of Evans Blue in the Evans Blue group and in the Evans Blue+iRGD group was higher than that in the PBS or iRGD group (Fig 2C). The amount of Evans Blue in the tumors from the Evans Blue+iRGD group was 1.5 times higher than that in the Evans Blue group (Fig 2C). With respect to the other organs, no apparent difference was observed between the Evans Blue group and the Evans Blue+iRGD group (Fig 2C). These data demonstrated that iRGD has the potential to specifically promote the uptake of drugs and allow them to penetrate into tumor.

### Therapeutic efficacy of Gemcitabine co-administered with iRGD

To determine the appropriate concentration of Gemcitabine for the in vivo experiments, four doses of Gemcitabine (80 mg/kg, 100 mg/kg, 120 mg/kg and 140 mg/kg) were used to treat four group mice twice one week for two weeks, respectively. Two weeks later, the mice of 80 mg/kg and 100 mg/kg groups appeared normal. However, it was apparent that the mice of 120 mg/kg group lacked energy and experienced a loss of appetite. Additionally, the mice of 140 mg/kg group had diarrhea. To reduce the impact of the side effects, we chose the dose of 100 mg/kg for in vivo experiments.

**Fig 3:**
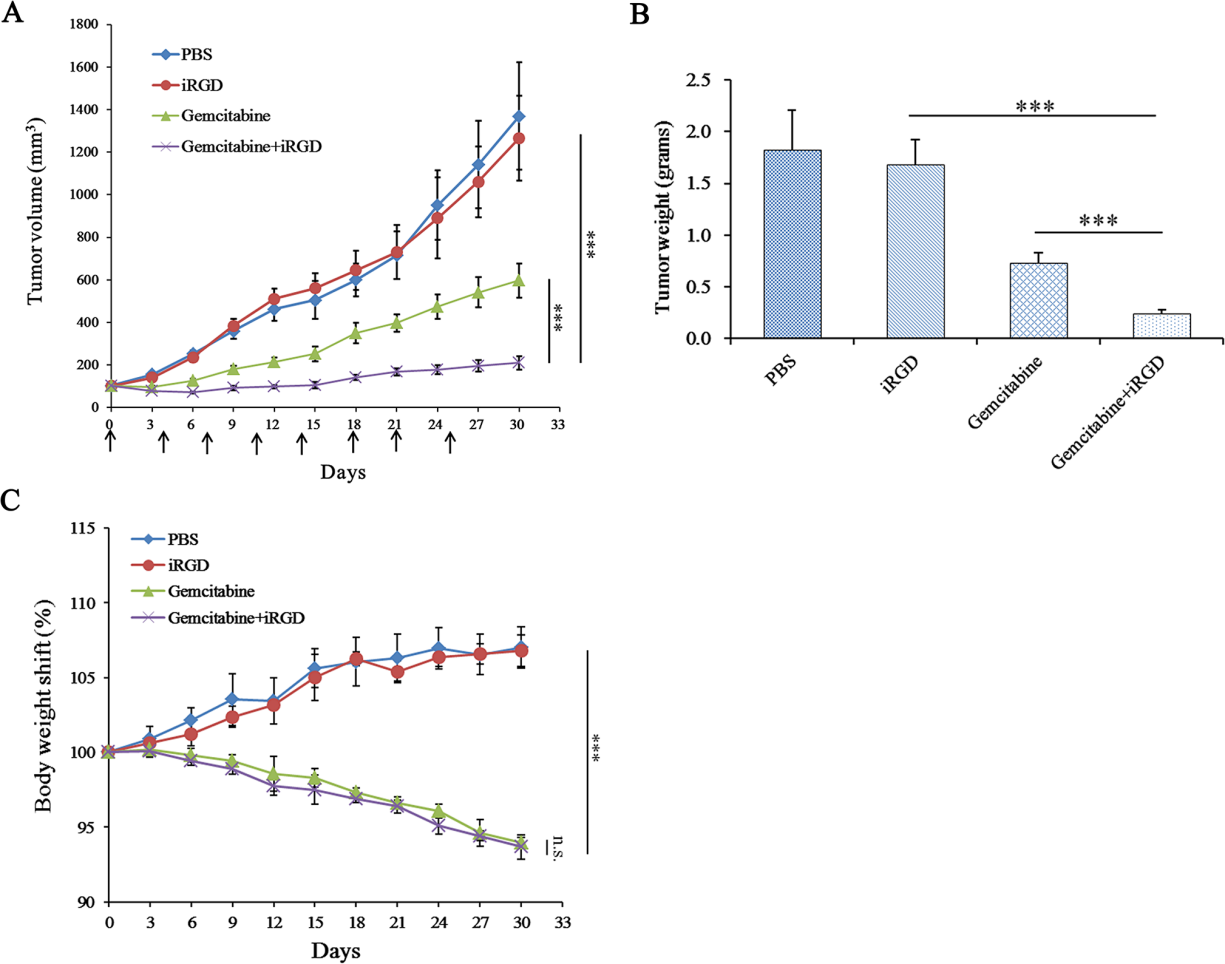
Therapeutic efficacy of Gemcitabine combined with iRGD for human NSCLC xenografts. (A) The tumor volume curve during treatment. Arrows indicate the time of injection. The day when treatment started was recorded as day 0. Tumor volume was measured once every three days until day 30. (B) Average tumor weight of each group at the end of treatment. (C) The body weight shift curve of the mice during the experiment. n = 6. Error bars, mean ± SD; ns, not significant; *** *p* < 0.001.

To determine whether the therapeutic efficacy of Gemcitabine will be enhanced when given in combination with iRGD, Gemcitabine and iRGD were co-administered to treat the mice with A549 xenografts as described in the Methods section. As shown in Fig 3A, the tumors of the Gemcitabine+iRGD group grew more slowly than those of the Gemcitabine group, and this difference was statistically significant. This observation was also confirmed by an analysis of the tumor weight (Fig 3B). The rate of growth inhibition of the tumors was further analyzed. The iRGD group, the Gemcitabine group, and the Gemcitabine+iRGD group showed a tumor growth inhibition rate of 8%, 59.8% and 86.9%, respectively. These results demonstrated that iRGD could enhance the therapeutic efficacy of Gemcitabine for human NSCLC xenografts when they were co-administered. The body weight shift analysis of the experimental mice showed that the body weight of the mice in both the Gemcitabine group and the Gemcitabine+iRGD group declined approximately 5% at the end of the experiment. However, no significant difference was observed between these two groups (Fig 3C). These results demonstrated that iRGD did not obviously exacerbate the side effects of Gemcitabine.

### The inhibition of cell proliferation after treatment with co-administered Gemcitabine and iRGD

**Fig 4:**
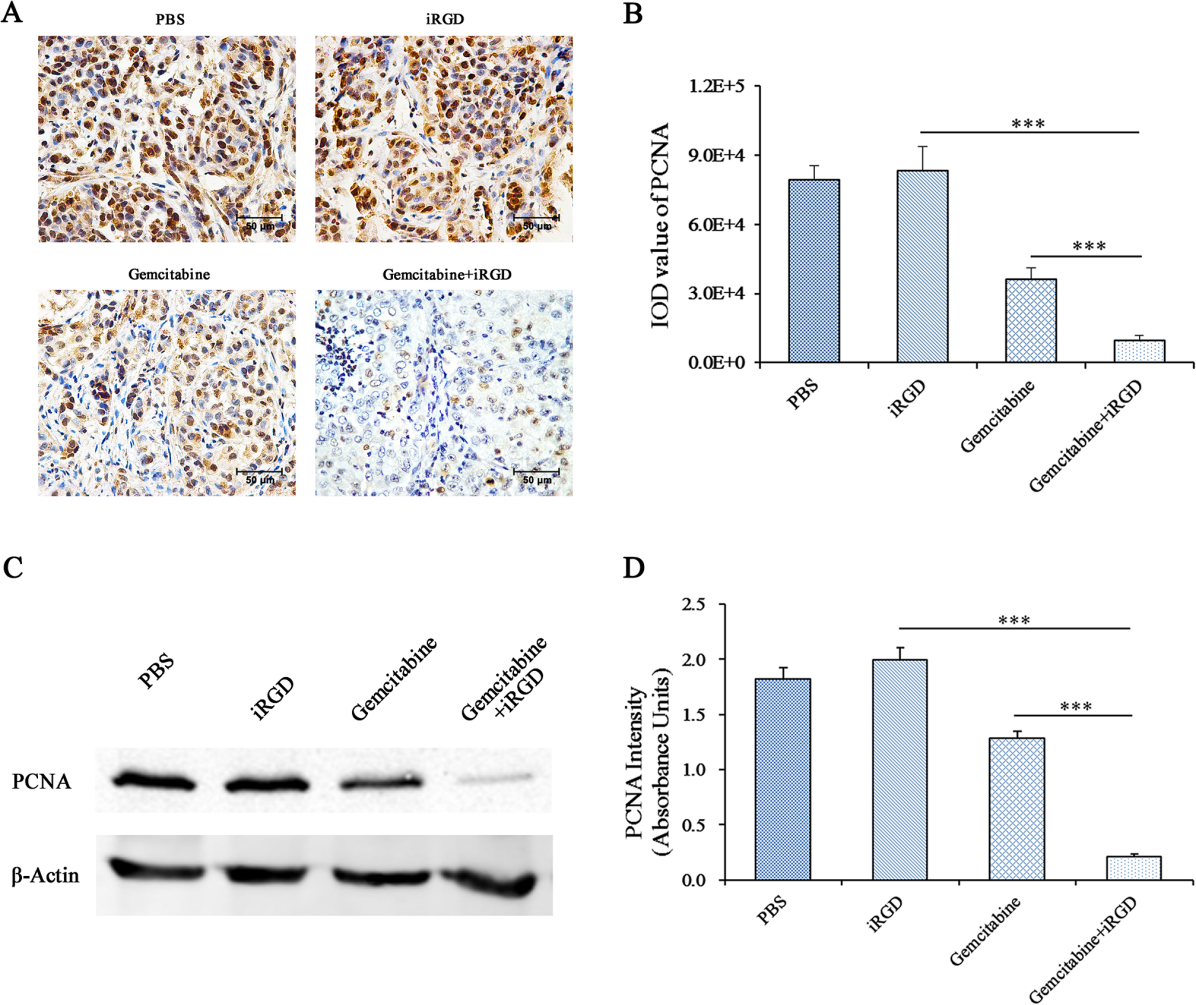
The expression of PCNA in tumor tissue. (A) Immunohistochemical staining analysis. PCNA-positive cells in the sections are stained brown. The nuclei are stained blue. Representative figures from each group are shown; n = 6; Magnification, × 400; Scale bars = 50 μm. (B) The corresponding quantitative analysis results of PCNA are shown in (A). Five fields from each tumor tissue section were randomly selected for the calculation of the IOD value of the positive region through IPP software, which indicated the amount of antigen expression. (C) Western blot analysis. Total protein from the tumor tissues was extracted. PCNA was detected with β-actin as an internal control. n = 3. (D) The quantitative analysis results of PCNA are shown in (D). The intensity of each strip was analyzed by ImageJ software. The average intensities of PCNA were standardized to β-actin. Error bars, mean ± SD; ns, not significant; *** *p* <0.001.

Gemcitabine exerts a therapeutic effect against cancer via the inhibition of the DNA synthesis in the cancer cells and interruption of their progress from G1 to S phase [3, 29, 30]. PCNA, a 36-kDa protein, is mainly expressed during S phase and early G2 phase of the cell cycle in proliferating cells [31]. Therefore, PCNA can be used as a marker of cell proliferation. To further confirm the function of iRGD in the enhancement of the anti-tumor effect of Gemcitabine, the expression level of PCNA in the tumors was detected by immunohistochemical staining. As shown in Fig 4A, the expression level of PCNA was significantly reduced in mice that were treated with Gemcitabine (with or without iRGD) compared to the expression level in mice who received PBS or iRGD; the expression level of PCNA was lower in the mice that were treated with Gemcitabine+iRGD compared with mice that were treated with Gemcitabine alone. For the quantitative analysis of Fig 4A, the expression level of PCNA in the Gemcitabine+iRGD group was reduced approximately 71.5% compared with that in the Gemcitabine group, and this difference was statistically significant (Fig 4B). In addition, the tumor tissue of Gemcitabine+iRGD group showed structural anomalies in cancer nests and morphological changes in most nuclei. The morphological changes in the nuclei will be described in detail later in the manuscript.

To further confirm the expression level of PCNA, we extracted the total protein from the tumor tissues in each group. The levels of PCNA were detected by Western blotting with β-actin as an internal control. Each strip was scanned, and a quantitative analysis was performed with ImageJ software. The expression level of PCNA in the tumor tissue of mice in the Gemcitabine+iRGD group was significantly reduced compared with that of mice in the Gemcitabine group (Fig 4C and 4D).

Together, these results further confirmed that the therapeutic efficacy of Gemcitabine was enhanced when it was co-administered with the tumor-penetrating peptide iRGD.

### Induction of apoptosis by co-administered Gemcitabine and iRGD

**Fig 5:**
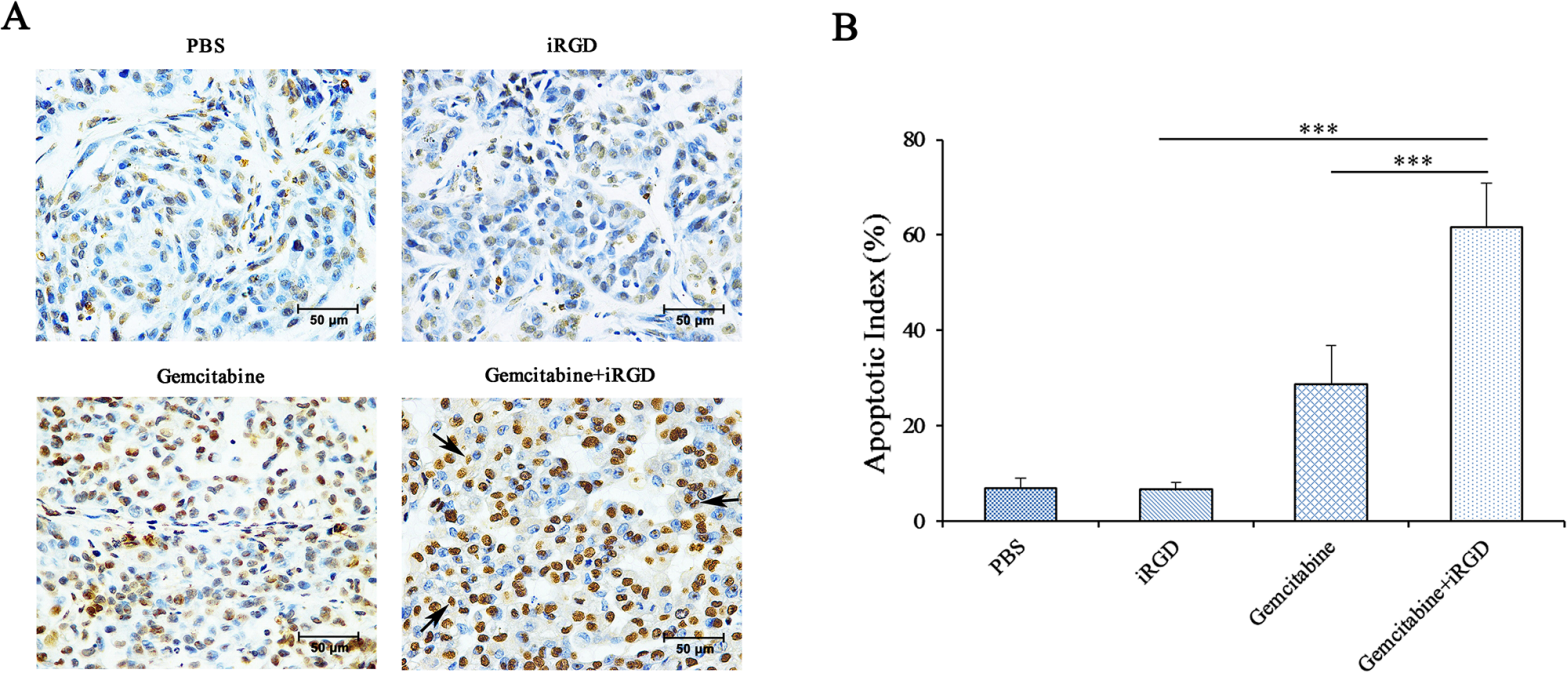
Induction of apoptosis by Gemcitabine combined with iRGD. (A) Apoptotic cells in the tumor tissues were detected by a TUNEL assay. TUNEL-positive nuclei are stained brown, and TUNEL-negative nuclei are stained blue. The figures shown here are representative of the six tumors in each group. Arrows indicate the apoptotic bodies. Magnification, × 400; Scale bars = 50 μm. (B) Quantitative analysis of the apoptosis index in each group. The percentage of TUNEL-positive cells was counted from 100 randomly selected tumor cells per section. Five sections were counted per tumor. n = 6; Error bars, mean ± SD; *** *p* < 0.001.

It has been shown that Gemcitabine can inhibit DNA replication and repair, which ultimately leads to cell apoptosis [3]. To confirm the enhanced therapeutic efficacy of co-administered Gemcitabine and iRGD, apoptotic cells in treated xenografts were detected using a TUNEL assay. As shown in Fig 5A, more apoptotic cells were visible in tumors from the Gemcitabine+iRGD group than in tumors from the Gemcitabine group. The apoptotic index was defined as the percentage of TUNEL-positive cells versus the total number of cells. According to the statistical analysis shown in Fig 5B, the rate of apoptosis of the Gemcitabine+iRGD group was 2.2 time that of the Gemcitabine group. These results further confirmed that iRGD enhanced the therapeutic efficacy of Gemcitabine in the NSCLC xenografts.

## Discussion

This study aimed to re-evaluate the use of iRGD peptide, demonstrated that it enhanced the accumulation and therapeutic efficacy of drugs in the NSCLC xenograft established with A549 cell line that showed high expression of αvβ3, αvβ5 and NRP-1. Our data showed that iRGD was able to significantly boost the tumor-penetration of Evans Blue as well as the therapeutic efficacy of Gemcitabine in the human NSCLC xenograft. The further evaluation showed that the proliferation and apoptosis of tumor cells in the iRGD+Gemcitabine group was more significantly inhibited than that in control groups.

In 2009, Teesalu etal. first reported the C-end rule peptide (iRGD), and demonstrated that the peptide possesses the activity of NRP-1-dependent cell, vascular, and tissue penetration [32]. Subsequent reports showed that iRGD could enhance the intratumoral dissemination and antitumor efficacy of Doxorubicin [33–35], Paclitaxel [36, 37] and Endostatin [38] by conjugating with these drugs or drug-loaded vehicles in animal experiments. The other reports demonstrated that iRGD also could enhance the intratumoral accumulation and antitumor efficacy of Paclitaxel, Doxorubicin, Transtuzumab [24, 39], Cisplatin [40], by co-administration with these drugs. In 2014, Akashi etal. reported that Gemcitabine was coadministrated with iRGD to treat pancreatic cancer in murine models [41]. To our knowledge, this study firstly demonstrated the efficacy of co-administrated iRGD and Gemcitabine in the human NSCLC xenograft established with A549 cell line.

Akashi’s report showed that pancreatic cancer models over-expressing NRP-1 are sensitive to iRGD co-administration. Treatment with Gemcitabine plus iRGD peptide resulted in a significant tumor reduction compared with Gemcitabine monotherapy in the pancreatic cancer models established with cell lines [41]. Our results showed that ανβ3, ανβ5 and NRP-1, which mediate the tumor-penetration activity of iRGD, were overexpressed in human NSCLC cell line A549 and xenograft established with this cell line. The therapeutic efficacy of Gemcitabine was significantly enhanced by co-administered iRGD in murine NSCLC cancer model. Together, these studies confirmed the effects of iRGD to enhance the intratumoral dissemination and anticancer efficacy in NRP-1-overexpressed cancer models.

Gemcitabine is a nucleoside analogue widely used as the first-line chemotherapy of advanced NSCLC [42]. Gridelli etal. reported that the response rate (RR) among 233 patients received Gemcitabine was 16%, whereas the median time to progression and overall survival (OS) were 17 weeks and 28 weeks, respectively [43]. The MILES-2G phase 2 study of single-agent Gemcitabine in advanced NSCLC elderly patients showed a 17.6% RR, a median time to disease progression of 16.1 weeks and median OS of 41.3 weeks [44]. Several other studies employing Gemcitabine alone showed same activity with RR and median PFS and OS ranging from 16–38.5%, 3–5.4 and 6.6–7.7 months, respectively [45–48]. Therefore, the therapeutic efficacy of Gemcitabine for NSCLC has the space to be further improved. Our results showed that iRGD peptide enhances the effects of co-administered Gemcitabine in the NSCLC xenograft. These results demonstrated the possibility that iRGD improves the effect of Gemcitabine in NSCLC patients.

However, the efficacy of iRGD is only appreciable in the employing cell line. The clinical application needs to be given careful consideration. The current understanding of the mechanism by which iRGD enhances the therapeutic efficacy of Gemcitabine for NSCLC is limited. Although no evident side effects were observed in our study, the safety of this combination therapy still needs further evaluation. The CendR motif of iRGD may also be used by viruses and microbial toxins in order to gain entry into cells and spread within the tissues of the body [49, 50]. iRGD also may promote the uptake of Gemcitabine into normal tissues that are damaged, and the tissue repair that follows may cause further damage to the body.

## Conclusion

In summary, through a human NSCLC A549 nude mouse xenograft model, it was confirmed that iRGD can promote the inhibition of tumor cell proliferation and the induction of apoptosis by Gemcitabine and therefore enhance the therapeutic efficacy of Gemcitabine in vivo. The co-administration of Gemcitabine and iRGD may be a novel strategy to enhance the clinical therapeutic efficacy of Gemcitabine in NSCLC patients.
